# Great differences in performance and outcome of high-throughput sequencing data analysis platforms for fungal metabarcoding

**DOI:** 10.3897/mycokeys.39.28109

**Published:** 2018-09-11

**Authors:** Sten Anslan, R. Henrik Nilsson, Christian Wurzbacher, Petr Baldrian, Leho Tedersoo, Mohammad Bahram

**Affiliations:** 1 Braunschweig University of Technology, Zoological Institute, Mendelssohnstr. 4, 38106 Braunschweig, Germany; 2 Gothenburg Global Biodiversity Centre, Department of Biological and Environmental Sciences, University of Gothenburg, Box 461, 405 30 Gothenburg, Sweden; 3 Technical University of Munich, Am Coulombwall 3, 85748 Garching, Germany; 4 Institute of Microbiology of the Czech Academy of Sciences, Videnska 1083, 14220 Praha 4, Czech Republic; 5 Natural History Museum of Tartu University, 14a Ravila, 50411 Tartu, Estonia; 6 Institute of Ecology and Earth Science, Tartu University, 14a Ravila, 50411 Tartu, Estonia; 7 Department of Organismal Biology, Evolutionary Biology Centre, Uppsala University, Norbyvägen 18D, Uppsala, Sweden; 8 Department of Ecology, Swedish University of Agricultural Sciences, Ulls väg 16, 756 51 Uppsala, Sweden

**Keywords:** Microbial communities, microbiome, mycobiome, fungal biodiversity, metagenomics, amplicon sequencing

## Abstract

Along with recent developments in high-throughput sequencing (HTS) technologies and thus fast accumulation of HTS data, there has been a growing need and interest for developing tools for HTS data processing and communication. In particular, a number of bioinformatics tools have been designed for analysing metabarcoding data, each with specific features, assumptions and outputs. To evaluate the potential effect of the application of different bioinformatics workflow on the results, we compared the performance of different analysis platforms on two contrasting high-throughput sequencing data sets. Our analysis revealed that the computation time, quality of error filtering and hence output of specific bioinformatics process largely depends on the platform used. Our results show that none of the bioinformatics workflows appears to perfectly filter out the accumulated errors and generate Operational Taxonomic Units, although PipeCraft, LotuS and PIPITS perform better than QIIME2 and Galaxy for the tested fungal amplicon dataset. We conclude that the output of each platform requires manual validation of the OTUs by examining the taxonomy assignment values.

## Introduction

Fungi are major ecological and functional players in terrestrial ecosystems. The full diversity of fungi remains largely uncharted due to their largely unculturable nature, the lack of tangible morphological manifestations and shortcomings of the mycological community to sample beyond traditional habitats and substrates (Grossart et al. 2016; Hibbett et al. 2017; Lücking et al. 2018). As a result, identification of fungi has come to rely mainly on direct DNA sequencing of material containing fungal hyphae or spores. In this regard, several DNA barcoding regions have been evaluated and the current consensus is that the nuclear ribosomal internal transcribed spacer (ITS) region is the best region for delimiting fungal taxa at the species level across a variety of fungal groups (Schoch et al. 2012). Recent advances in high-throughput sequencing (HTS) have made it possible to sequence millions of reads and identify thousands of fungal taxa from a single sample. Handling this enormous amount of data is often complicated and requires extensive bioinformatics expertise.

Multiple analysis platforms have been introduced to facilitate the bioinformatics treatment of HTS data. However, most of these software suites were developed for the prokaryotic 16S rRNA gene and may therefore perform poorly with other markers and other organisms, in particular ITS sequences due to their length variation and non-alignability across taxonomic expanses. To accommodate this, several tailored platforms have recently been developed to specifically address fungal ITS datasets (Anslan et al. 2017; Gweon et al. 2015; Hildebrand et al. 2014; Vetrovský et al. 2018). These platforms cover multiple steps of the analysis procedure, including quality control, clustering, taxonomic assignment and generating Operational Taxonomic Unit (OTU) abundance tables. Many of these platforms cover all these analysis steps, whereas others do not.

The application of different bioinformatics workflows may introduce variation in the data quality and output OTU tables (Majaneva et al. 2015; Sinha et al. 2017). However, to date, there are no data on the relative performance of the available tools for fungal HTS data analysis. In this study, we report on the relative performance of the most popular software pipelines on two contrasting HTS datasets.

## Methods

### Sequence data and general workflow

**Figure 1.**
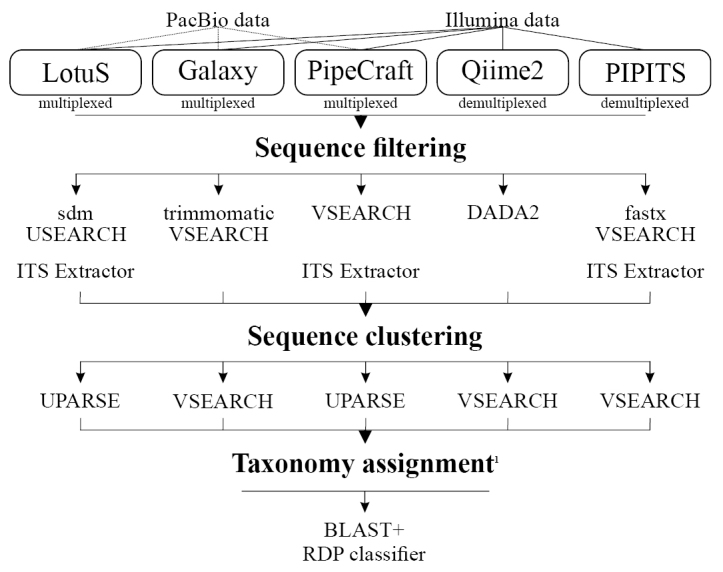
Outline of workflow in different analysis pipelines.

We compared the performance of bioinformatics analysis platforms on two fungal ITS datasets. Tested datasets included Illumina MiSeq paired-end ITS2 amplicons from arthropod substrates (Anslan et al. 2018) and full ITS circular consensus sequences from Pacific Biosciences (PacBio) Sequel machine, amplified from soil samples. PacBio data set is available through PlutoF database (Abarenkov et al. 2010b), https://plutof.ut.ee/#/datacite/10.15156%2FBIO%2F781236). For bioinformatics analyses, we used multiple platforms that support all steps in the analysis of HTS-based metabarcoding datasets: QIIME2 (v2018.2; Caporaso et al. 2010), LotuS (v1.59; Hildebrand et al. 2014), Galaxy (v.2.1.1; Afgan et al. 2016), PipeCraft (v1.0; Anslan et al. 2017) and PIPITS (v2.0; Gweon et al. 2015) (Table 1; Figure 1). Depending on the analysis platform, quality filtering was performed using either VSEARCH (Rognes et al. 2016), trimmomatic (Bolger et al. 2014), DADA2 (Callahan et al. 2016), sdm (Hildebrand et al. 2014) or fastx (http://hannonlab.cshl.edu/fastx_toolkit). Quality filtered sequences were passed through chimeric reads removal algorithms as implemented in USEARCH (Edgar 2013; Edgar et al. 2011) or VSEARCH. Using PipeCraft, LotuS and PIPITS, reads were also subjected to ITS extraction using ITSx (Bengtsson-Palme et al. 2013) to remove conservative flanking genes of the ITS region. OTU formation (clustering) was performed using USEARCH or VSEARCH as outlined below (Platform specific options). For each platform, we relied on *de-novo* single linkage clustering, which is the most popular approach in fungal community studies, knowing that reference-based clustering methods can provide similar results (Cline et al. 2017). Taxonomic affiliations were assigned to OTUs using DP Naive Bayesian rRNA Classifier (RDP classifier v2.11; Wang et al. 2007) with the Warcup Fungal ITS trainset 2 (confidence threshold: 80%; Deshpande et al. 2016) as well as BLAST+ (Camacho et al. 2009) search (e-value = 0.001, word size = 7, reward = 1, penalty = -1, gap open = 1, gap extend = 2) against the UNITE v7.2 reference database (Abarenkov et al. 2010a).

**Table 1.** Used software, sequence and OTU counts (values in bold) by **a**) Illumina and **b**) PacBio analysis platforms. The number of sequences denotes raw input reads and remaining reads after each analysis step. Singleton OTUs were excluded from the OTU counts.

a)	LotuS	Qiime2	PipeCraft	Galaxy	PIPITS
Raw reads	7,981,812a	7,335,838b	7,981,812a	7,981,812a	7 335 838b
Assembly	FLASH/ NA	DADA2/ NA	VSEARCH/ 7,511,274	FASTQ joiner/ 7,911,554	VSEARCH/ 7,198,094
Quality filtering	sdm/NA	DADA2/ 5,428,563	VSEARCH/ 7,511,274	trimmomatic/ 7,879,960	fastqx/ 7,142,354
Demultiplexing	sdm/ 6,727,631	NP	mothur/ 6,558,772	mothur/ 1,643,879	NP
Chimera filtering	USEARCH/ 6,486,802	NP	VSEARCH/ 6,300,085	VSEARCH/ 1,621,330	VSEARCH/ NA
ITS extractor	5,919,084	NP	6,262,000	NP	6,401,097
Clustering (OTUs)	UPARSE/ 8,659	VSEARCH/ 7,477	UPARSE/ 7,598	VSEARCH/ 23,167	VSEARCH/ 7,887
**b)**	**LotusS**	**PipeCraft**	**Galaxy**		
CCSc reads	720,222a	720,222a	720,222a		
Quality filtering	sdm/ NA	VSEARCH/ 462,010	trimmomatic/ 672,292		
Demultiplexing	sdm/ 258,085	mothur/ 380,722	mothur/ 457,173		
Chimera filtering	USEARCH/ 255,746	VSEARCH/ 341,154	VSEARCH/ 405,025		
ITS extraction	192,485	338,150	NP		
Clustering (OTUs)	UPARSE/ 942	UPARSE/ 4,176	VSEARCH/ 8,338		

^a^multiplexed input data; ^b^demultiplexed input data; ^c^circular consensus sequences; NA: indicate not available; NP: not performed.

### Platform specific options

Using QIIME2, reads were assembled (Illumina data) and quality filtered using DADA2 (Callahan et al. 2016) with default options, except --p-trunc-len = 0, --p-max-ee = 1 and --p-chimera-method = none (with chimera-method = consensus, QIIME2 reported error for our data). Clustering was performed with VSEARCH cluster-features-de-novo (--p-perc-identity 0.97).

In LotuS pipline, data was assembled (Illumina data), quality filtered (minimum length = 170, minAvgQuality = 27, TruncateSequenceLength = 170, maxAccumulatedError = 0.75) and demultiplexed with sdm (pdiffs = 1, bdiffs = 1). Chimera filtering was undertaken using USEARCH *de novo* chimera filtering (abundance annotation = 0.97, abskew = 2) and USEARCH reference-based chimera filtering using UNITE v7.2 as reference database. Flanking genes of the ITS region were discarded using ITSx (v1.0.11; default options). ITS reads were clustered to OTUs with USEARCH/UPARSE algorithm (-id = 3, -minsize = 2).

Using web-based Galaxy pipeline, Illumina data were assembled with Fastq joiner (Galaxy Version 2.0.1.1; Blankenberg et al. 2010) with default options. Quality filtering was performed with Trimmomatic (Galaxy Version 0.36.3) ‒ SLIDINGWINDOW; number of bases to average across = 15, average quality required = 30, minimum length of kept reads = 45. Fastq files were converted to FASTA files using FASTQ to FASTA converter (Galaxy Version 1.0.0). Fasta files were demultiplexed using mothur (Galaxy Version 1.39.5.0; Schloss et al. 2009) ‒ pdiffs = 2, bdiffs = 1. As sequences were of mixed orientation in the files (5’-3’ and 3’-5’), the demultiplexing step was repeated for reverse orientated sequences (reads were reversed using mothur reverse.seqs). Chimera filtering was undertaken using VSEARCH chimera detection (Galaxy Version 1.9.7.0) with default settings (abundance annotation = 97% similarity threshold) and using the UNITE v7.2 database as reference. Clustering was performed using VSEARCH (--cluster-fast, --id 0.97, --iddef 1).

In PipeCraft, platform reads were assembled (Illumina data) and quality filtered using VSEARCH (minimum overlap = 15, minimum length = 100, E max = 1, max ambiguous = 0, allowstagger = T). Demultiplexing was undertaken using mothur (pdiffs = 2, bdiffs = 1). In this step, sequences are also re-orientated into the 5’-3’ orientation based on primers (2 mismatches allowed).

Chimeric sequences were removed using VSEARCH *de novo* (abundance annotation = 0.97, abskew = 2) and reference-based (UNITE v7.2 as reference) chimera filtering algorithms. In the chimera filtering step, the PipeCraft supported option for “primer artefact” removal was also used (sequences where primer strings were found in the middle of the sequence were removed). ITS reads were extracted using ITSx (default options). Clustering was performed using USEARCH/UPARSE algorithm (id = 3, minsize = 2).

Using PIPITS, sequences were assembled with VSEARCH and quality-filtering was undertaken with fastx through the PIPITS command pispino_createreadpairslist. The ITSx was executed through the PIPITS command pipits_funits. Chimera filtering and clustering were undertaken using VSEARCH through the PIPITS command pipits_process.

### Additional filtering

The additional manual OTU table filtering was based on the BLAST similarity scores when run against UNITE (v7.2) reference database. Any OTUs that had no BLAST hit or that were not classified to the kingdom Fungi were discarded from the OTU table. The remaining OTUs were filtered based on BLAST e-value and query coverage. OTUs with higher e-value than 1e^-25^ and query coverage less than 70% were excluded from the dataset (as putative artefacts or non-fungal OTUs). Additionally, OTUs with low numbers of sequences per sample were removed (less than 10 sequences per sample; Brown et al. (2015)). Finally, the LULU (Frøslev et al. 2017) algorithm was applied (minimum_ratio_type = “min”, minimum_match = 97) to merge consistently co-occurring ‘daughter’ OTUs.

### Data pooling

To detect the effect of analysis platform choice on the OTU composition, we pooled sequences originating from different platforms and applied the common clustering method to generate a single OTU table. For Illumina data, filtered reads from PipeCraft, LotuS and PIPITS were pooled and clustered using CD-HIT (Fu et al. 2012) at 97% sequence similarity (Table 1). The pooled PacBio dataset included filtered sequences from LotuS, PipeCraft and Galaxy platform, clustering was performed using UPARSE algorithm with 97% sequence similarity threshold (Table 1).

### Statistical analysis

We used PERMANOVA analysis (Anderson and Walsh 2013; Type III SS, 4,999 permutations) on Bray-Curtis distances of Hellinger-transformed OTU matrices, using PRIMER6 (Clarke and Gorley 2006). Outliers were screened and removed using analysis of non-metric multidimensional scaling (NMDS). The numbers of sequences per sample were included in the analysis as covariates. Rarefaction curves were generated based on OTU abundance matrices for each dataset using the RTK package (Saary et al. 2017) of R (R-Core-Team 2015).

## Results and discussion

### Properties of bioinformatics analysis platforms

All tested bioinformatics platforms offer straightforward installation. While Galaxy provides a freely available online platform, the benefits of PipeCraft and QIIME2 include easy-to-use graphical user interfaces and multiple options for data analysis. These platforms bundle many tools for diverse tasks. LotuS and PIPITS represent command-line based platforms. PIPITS offers a limited number of tools, but data analysis is easily performed with a straightforward pipeline. LotuS has been developed to minimise computational time and memory requirements. Specifically, for accuracy of ITS-based analyses of fungi and other eukaryotes, PipeCraft, LotuS and PIPITS implement the ITSx tool (Bengtsson-Palme et al. 2013), which removes the fragments of conservative flanking genes for precise clustering purposes. There is no such option in QIIME2 and Galaxy.

Bioinformatics platforms differ by specific requirements to the input data, with the options being a raw multiplexed file (a single file containing all sequences from one run) and multiple demultiplexed files (reads split into separate files based on indexes). PipeCraft and Galaxy use raw multiplexed data, whereas QIIME2 and PIPITS require demultiplexed files. Only LotuS allows both, multiplexed and demultiplexed files as input. As the raw data files are multiplexed by default, QIIME2 and PIPITS platforms required additional steps of analyses outside these tools to meet the input requirements. Using a Python script, we demultiplexed the raw Illumina data, allowing 2 and 1 mismatches to primer and index strings, respectively. However, PacBio data analysis was dropped for QIIME2 and PIPITS as the present versions of these platforms are limited to analysis of short read (Illumina) data.

### Performance of bioinformatics platforms on sequence data

**Figure 2.**
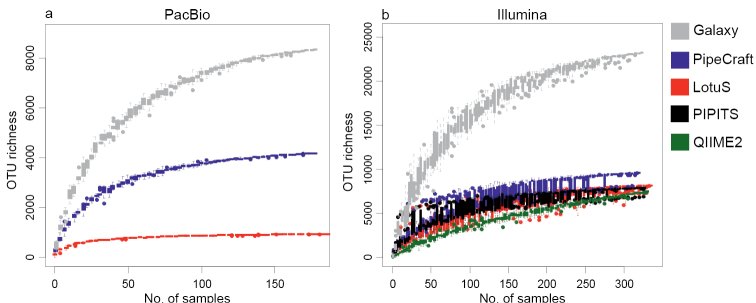
OTU accumulation curves of the evaluated pipelines for a) PacBio and b) Illumina datasets.

**Figure 3.**
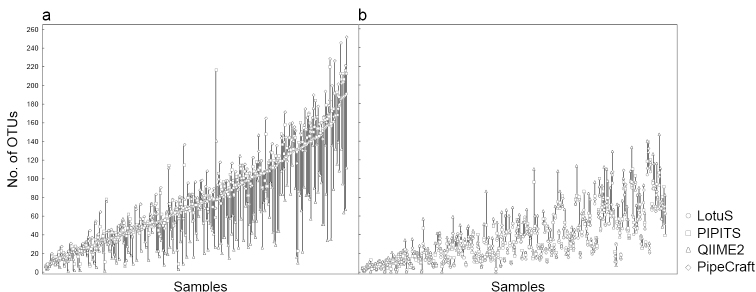
Number of OTUs per sample for Illumina data recorded from a) pipeline-generated OTU tables (median differences = 38 OTUs) and from b) filtered OTU tables (median differences = 12 OTUs). The Galaxy workflow was excluded here.

For both the Illumina and PacBio datasets, the final OTU richness (singleton OTUs excluded) differed considerably amongst the tested workflows (Table 1). We found that pipelines, which produced roughly comparable numbers of total OTUs (QIIME2, PipeCraft, PIPITS and LotuS for Illumina data), still exhibited large variations in OTU richness per sample (Figures 2 and 3). By performing joint *de-novo* clustering for filtered sequences from different pipelines (total number of OTUs = 16333), we observed a weak but significant effect of pipeline choice on overall OTU composition for the Illumina data set (PERMANOVA: pseudo-F_2,868_ = 5.88, R^2^_adj_ = 0.012, P < 0.001). For the PacBio dataset (total number of OTUs = 4448), differences amongst platforms were slightly stronger (pseudo-F_2,512_ = 9.174; R^2^_adj_ = 0.033, P < 0.001).

Taxonomic annotation tools differed in the ability to classify OTUs. In general, BLAST searches revealed many cases of high-quality matches to non-fungal organisms (in some cases for hundreds of OTUs), while RDP when combined with the Warcup Fungal ITS trainset optimistically classified all OTUs to Fungi (100% confidence). Numerous papers have evaluated the performance of different methods on the accuracy of taxonomic assignment and performance inevitably hinges on the completeness of the reference database used (e.g. Gdanetz et al. 2017; Richardson et al. 2017). In spite of its relatively rapid performance, the RDP Fungal ITS trainset does not include any non-fungal data, which explains its shortcomings in detecting non-fungal OTUs. However, the confidence score of an RDP classifier did not exceed 64% for non-fungal OTUs, mostly overestimating the group of unclassified fungi.

We also observed that the quality-filtered datasets included up to ~10% of obvious erroneous/chimeric OTUs that produced matches with low query coverage and confidence scores. A long tail of satellite OTUs, assigned to a single species hypothesis with 99–100% BLAST identity and RDP classifier confidence level, were also common – especially in the results where a relatively high number of OTUs was observed (Galaxy platform). After filtering the spurious OTUs manually (see Methods), we found that richness estimates per sample became more homogeneous across pipelines (Illumina data: Figure 3). When OTU table filtering was applied to jointly clustered reads from different pipelines, the significant effect of pipeline choice on the community composition diminished (Illumina data: pseudo-F_2,837_ = 0.955, R^2^_adj_ = 0.007, P = 0.779).

In conclusion, our results indicate that bioinformatics analysis pipelines greatly differ in their relative performance on ITS datasets targeting fungi, although roughly similar quality-orientated settings were implemented. Overall, our recommended Illumina data workflow would be PipeCraft, PIPITS or LotuS, which provide a good balance between speed, mycological accuracy (including support for ITS Extractor) and technical quality. For PacBio, the tools implemented in PipeCraft were most suitable for the long-read analysis. Conversely, the widely used platform in prokaryote 16S-based studies, our options chosen in Galaxy, performed relatively poorly on the ITS data. While QIIME2 implements an accurate quality filtering algorithm of DADA2, the lack of ITS region extraction lowers the accuracy for mycological studies. Of classification tools, BLAST searches against the UNITE database provided more accurate results on the kingdom and phylum levels compared with the RDP and Warcup ITS trainset combined. We emphasise that none of the tested bioinformatics workflows is able to fully filter out the errors that accumulated during sample preparation and sequencing, even when using the most elaborate error-filtering options. Therefore, manual curation of OTU tables continues to be an important step in obtaining robust datasets, although semi-automatic tools to assist evaluation are becoming available (Frøslev et al. 2017). It is also important to rely on high-coverage reference databases to be able to recognise non-target organisms and metagenomic reads.

## References

[B1] Abarenkov K Nilsson RH Larsson K-H Alexander IJ Eberhardt U Erland S Hoiland K Kjoller R Larsson E Pennanen T Sen R Taylor AFS Tedersoo L Ursing BM Vralstad T Liimatainen K Peintner U Kõljalg U (2010a) The UNITE database for molecular identification of fungi – recent updates and future perspectives. New Phytologist 186: 281–285. 10.1111/j.1469-8137.2009.03160.x 20409185

[B2] Abarenkov K Tedersoo L Nilsson RH Vellak K Saar I Veldre V Parmasto E Prous M Aan A Ots M Kurina O Ostonen I Jogeva J Halapuu S Poldmaa K Toots M Truu J Larsson K-H Koljalg U (2010b) PlutoF-a Web Based Workbench for Ecological and Taxonomic Research, with an Online Implementation for Fungal ITS Sequences. Evolutionary Bioinformatics 6: 189–196. 10.4137/ebo.s6271

[B3] Afgan E Baker D Van den Beek M Blankenberg D Bouvier D Čech M Chilton J Clements D Coraor N Eberhard C (2016) The Galaxy platform for accessible, reproducible and collaborative biomedical analyses: 2016 update. Nucleic Acids Research 44: 3–10. 10.1093/nar/gkw343 PMC4987906 27137889

[B4] Anderson MJ Walsh DCI (2013) PERMANOVA, ANOSIM, and the Mantel test in the face of heterogeneous dispersions: What null hypothesis are you testing? Ecological Monographs 83: 557–574. 10.1890/12-2010.1

[B5] Anslan S Bahram M Hiiesalu I Tedersoo L (2017) PipeCraft: flexible open-source toolkit for bioinformatics analysis of custom high-throughput amplicon sequencing data. Molecular Ecology Resources 17: e234–e240. 10.1111/1755-0998.12692 28544559

[B6] Anslan S Bahram M Tedersoo L (2018) Seasonal and annual variation in fungal communities associated with epigeic springtails (Collembola spp.) in boreal forests. Soil Biology and Biochemistry 116: 245–252. doi:10.1016/j.soilbio.2017.10.021

[B7] Bengtsson-Palme J Ryberg M Hartmann M Branco S Wang Z Godhe A De Wit P Sanchez-Garcia M Ebersberger I de Sousa F Amend AS Jumpponen A Unterseher M Kristiansson E Abarenkov K Bertrand YJK Sanli K Eriksson KM Vik U Veldre V Nilsson RH (2013) Improved software detection and extraction of ITS1 and ITS2 from ribosomal ITS sequences of fungi and other eukaryotes for analysis of environmental sequencing data. Methods in Ecology and Evolution 4: 914–919. 10.1111/2041-210x.12073

[B8] Blankenberg D Gordon A Von Kuster G Coraor N Taylor J Nekrutenko A Team G (2010) Manipulation of FASTQ data with Galaxy. Bioinformatics 26: 1783–1785. 10.1093/bioinformatics/btq281 20562416 PMC2894519

[B9] Bolger AM Lohse M Usadel B (2014) Trimmomatic: a flexible trimmer for Illumina sequence data. Bioinformatics 30: 2114–2120. 10.1093/bioinformatics/btu170 24695404 PMC4103590

[B10] Brown SP Veach AM Rigdon-Huss AR Grond K Lickteig SK Lothamer K Oliver AK Jumpponen A (2015) Scraping the bottom of the barrel: are rare high throughput sequences artifacts? Fungal Ecology 13: 221–225. 10.1016/j.funeco.2014.08.006

[B11] Callahan BJ McMurdie PJ Rosen MJ Han AW Johnson AJA Holmes SP (2016) DADA2: high-resolution sample inference from Illumina amplicon data. Nature Methods 13: 581. 10.1038/nmeth.3869 PMC4927377 27214047

[B12] Camacho C Coulouris G Avagyan V Ma N Papadopoulos J Bealer K Madden TL (2009) BLAST+: architecture and applications. BMC Bioinformatics 10: 421. 10.1186/1471-2105-10-421 PMC2803857 20003500

[B13] Caporaso JG Kuczynski J Stombaugh J Bittinger K Bushman FD Costello EK Fierer N Peña AG Goodrich JK Gordon JI (2010) QIIME allows analysis of high-throughput community sequencing data. Nature Methods 7: 335–336. 10.1038/nmeth.f.303 20383131 PMC3156573

[B14] Clarke K Gorley R (2006) PRIMER V6: User Manual / Tutorial. Primer-E Ltd, Plymouth, 192 pp.

[B15] Cline LC Song Z Al‐Ghalith GA Knights D Kennedy PG (2017) Moving beyond de novo clustering in fungal community ecology. New Phytol. 216(3): 629–634. 10.1111/nph.14752 28782807

[B16] Deshpande V Wang Q Greenfield P Charleston M Porras-Alfaro A Kuske CR Cole JR Midgley DJ Tran-Dinh N (2016) Fungal identification using a Bayesian classifier and the Warcup training set of internal transcribed spacer sequences. Mycologia 108: 1–5. 10.3852/14-293 26553774

[B17] Edgar RC (2013) UPARSE: highly accurate OTU sequences from microbial amplicon reads. Nature Methods 10. 10.1038/nmeth.2604 23955772

[B18] Edgar RC Haas BJ Clemente JC Quince C Knight R (2011) UCHIME improves sensitivity and speed of chimera detection. Bioinformatics 27: 2194–2200. 10.1093/bioinformatics/btr381 21700674 PMC3150044

[B19] Frøslev TG Kjøller R Bruun HH Ejrnæs R Brunbjerg AK Pietroni C Hansen AJ (2017) Algorithm for post-clustering curation of DNA amplicon data yields reliable biodiversity estimates. Nature communications 8: 1188. 10.1038/s41467-017-01312-x PMC5662604 29084957

[B20] Fu L Niu B Zhu Z Wu S Li W (2012) CD-HIT: accelerated for clustering the next-generation sequencing data. Bioinformatics 28: 3150–3152. 10.1093/bioinformatics/bts565 23060610 PMC3516142

[B21] Grossart H-P Wurzbacher C James TY Kagami M (2016) Discovery of dark matter fungi in aquatic ecosystems demands a reappraisal of the phylogeny and ecology of zoosporic fungi. Fungal Ecology 19: 28–38. doi:10.1016/j.funeco.2015.06.004

[B22] Gweon HS Oliver A Taylor J Booth T Gibbs M Read DS Griffiths RI Schonrogge K (2015) PIPITS: an automated pipeline for analyses of fungal internal transcribed spacer sequences from the Illumina sequencing platform. Methods in Ecology and Evolution 6: 973–980. 10.1111/2041-210x.12399 27570615 PMC4981123

[B23] Hibbett D Abarenkov K Koljalg U Opik M Chai B Cole JR Wang Q Crous PW Robert VARG Helgason T Herr J Kirk P Lueschow S O’Donnell K Nilsson H Oono R Schoch CL Smyth C Walker D Porras-Alfaro A Taylor JW Geiser DM (2017) Sequence-based classification and identification of Fungi. Mycologia 108: 1049–1068 10.3852/16-130 27760854

[B24] Hildebrand F Tadeo R Voigt AY Bork P Raes J (2014) LotuS: an efficient and user-friendly OTU processing pipeline. Microbiome 2: 30. 10.1186/2049-2618-2-30 PMC4179863 27367037

[B25] Lücking R Kirk PM Hawksworth DL (2018) Sequence-based nomenclature: a reply to Thines et al. and Zamora et al. and provisions for an amended proposal. IMA fungus 9: 185–198. 10.5598/imafungus.2018.09.01.12 30018879 PMC6048568

[B26] Majaneva M Hyytiäinen K Varvio SL Nagai S Blomster J (2015) Bioinformatic amplicon read processing strategies strongly affect eukaryotic diversity and the taxonomic composition of communities. PLoS ONE 10: e0130035. 10.1371/journal.pone.0130035 PMC4457843 26047335

[B27] R-Core-Team (2015) R: A language and environment for statistical computing. R Foundation for Statistical Computing, Vienna.

[B28] Rognes T Flouri T Nichols B Quince C Mahé F (2016) VSEARCH: a versatile open source tool for metagenomics. PeerJ 4: e2584. 10.7717/peerj.2584 PMC5075697 27781170

[B29] Saary P Forslund K Bork P Hildebrand F (2017) RTK: efficient rarefaction analysis of large datasets. Bioinformatics 33: 2594–2595. 10.1093/bioinformatics/btx206 28398468 PMC5870771

[B30] Schloss PD Westcott SL Ryabin T Hall JR Hartmann M Hollister EB Lesniewski RA Oakley BB Parks DH Robinson CJ Sahl JW Stres B Thallinger GG Van Horn DJ Weber CF (2009) Introducing mothur: Open-Source, Platform-Independent, Community-Supported Software for Describing and Comparing Microbial Communities. Applied and Environmental Microbiology 75: 7537–7541. 10.1128/aem.01541-09 19801464 PMC2786419

[B31] Schoch CL Seifert KA Huhndorf S Robert V Spouge JL Levesque CA Chen W Bolchacova E Voigt K Crous PW Miller AN Wingfield MJ Aime MC An KD Bai FY Barreto RW Begerow D Bergeron MJ Blackwell M Boekhout T Bogale M Boonyuen N Burgaz AR Buyck B Cai L Cai Q Cardinali G Chaverri P Coppins BJ Crespo A Cubas P P Cummings C Damm U de Beer ZW de Hoog GS Del-Prado R Dentinger B Dieguez-Uribeondo J Divakar PK Douglas B Duenas M Duong TA Eberhardt U Edwards JE Elshahed MS Fliegerova K Furtado M Garcia MA Ge ZW Griffith GW Griffiths K Groenewald JZ Groenewald M Grube M Gryzenhout M Guo LD Hagen F Hambleton S Hamelin RC Hansen K Harrold P Heller G Herrera G Hirayama K Hirooka Y Ho HM Hoffmann K Hofstetter V Hognabba F Hollingsworth PM Hong SB Hosaka K Houbraken J Hughes K Huhtinen S Hyde KD James T Johnson EM Johnson JE Johnston PR Jones EB Kelly LJ Kirk PM Knapp DG Koljalg U Kovacs GM Kurtzman CP Landvik S Leavitt SD Liggenstoffer AS Liimatainen K Lombard L Luangsa-Ard JJ Lumbsch HT Maganti H Maharachchikumbura SS Martin MP May TW McTaggart AR Methven AS Meyer W Moncalvo JM Mongkolsamrit S Nagy LG Nilsson RH Niskanen T Nyilasi I Okada G Okane I Olariaga I Otte J Papp T Park D Petkovits T Pino-Bodas R Quaedvlieg W Raja HA Redecker D Rintoul T Ruibal C Sarmiento-Ramirez JM Schmitt I Schussler A Shearer C Sotome K Stefani FO Stenroos S Stielow B Stockinger H Suetrong S Suh SO Sung GH Suzuki M Tanaka K Tedersoo L Telleria MT Tretter E Untereiner WA Urbina H Vagvolgyi C Vialle A Vu TD Walther G Wang QM Wang Y Weir BS Weiss M White MM Xu J Yahr R Yang ZL Yurkov A Zamora JC Zhang N Zhuang WY Schindel D Fungal Barcoding C (2012) Nuclear ribosomal internal transcribed spacer (ITS) region as a universal DNA barcode marker for Fungi. Proceedings of the National Academy of Sciences of the United States of America 109: 6241–6246. 10.1073/pnas.1117018109 22454494 PMC3341068

[B32] Sinha R Abu-Ali G Vogtmann E Fodor AA Ren B Amir A Schwager E Crabtree J Ma S Abnet CC (2017) Assessment of variation in microbial community amplicon sequencing by the Microbiome Quality Control (MBQC) project consortium. Nature Biotechnology volume 35, pages 1077–1086. 10.1038/nbt.3981 PMC5839636 28967885

[B33] Wang Q Garrity GM Tiedje JM Cole JR (2007) Naive Bayesian classifier for rapid assignment of rRNA sequences into the new bacterial taxonomy. Applied and Environmental Microbiology 73. 10.1128/aem.00062-07 PMC1950982 17586664

[B34] Vetrovský T Baldrian P Morais D Berger B (2018) SEED 2: a user-friendly platform for amplicon high-throughput sequencing data analyses. Bioinformatics 1: 3. 10.1093/bioinformatics/bty071 PMC6022770 29452334

